# Effects of Spatial Characteristics on the Spread of the Highly Pathogenic Avian Influenza (HPAI) in Korea

**DOI:** 10.3390/ijerph18084081

**Published:** 2021-04-13

**Authors:** Meilan An, Jeffrey Vitale, Kwideok Han, John N. Ng’ombe, Inbae Ji

**Affiliations:** 1 Department of Food Industrial Management, Dongguk University, 30 Pildong-ro 1-gil, Jung-gu, Seoul 04620, Korea; zhensee@naver.com; 2 Department of Agricultural Economics, Oklahoma State University, 418 Ag Hall, Stillwater, OK 74078, USA; jeffrey.vitale@okstate.edu; 3 Department of Institutional Research and Analytics, Oklahoma State University, 203 PIO Building, Stillwater, OK 74078, USA; kwideok.han@okstate.edu; 4 Department of Agricultural Economics and Extension, University of Zambia, Lusaka 10101, Zambia; ngombe@okstate.edu

**Keywords:** highly pathogenic avian influenza (HPAI), regional characteristics, spatial dependence, spatial autoregressive model

## Abstract

This paper examines the effects of regional characteristics on the spread of the highly pathogenic avian influenza (HPAI) during Korea’s 2016–2017 outbreak. A spatial econometric model is used to determine the effects of regional characteristics on HPAI dispersion using data from 162 counties in Korea. Results indicate the existence of spatial dependence, suggesting that the occurrence of HPAI in a county is significantly influenced by neighboring counties. We found that larger size poultry, including laying hens, breeders, and ducks are significantly associated with a greater incidence of HPAI. Among poultry, we found ducks as the greatest source of the spread of HPAI. Our findings suggest that those regions that are spatially dependent with respect to the spread of HPAI, such as counties that intensively breed ducks, should be the focus of surveillance to prevent future epidemics of HPAI.

## 1. Introduction

The livestock industry in Korea has contributed to stabilizing farm household income in response to the substantial increase in Korean meat consumption that has occurred over the past couple of decades. However, increased incidence of livestock disease outbreaks, such as foot-and-mouth, Brucella, and highly pathogenic avian influenza (HPAI) has caused substantial production losses and economic damage to the livestock sector over the same period, thereby substantially threatening the emerging livestock sector in Korea [1]. Particularly, the spread of HPAI in Korea has largely varied across counties over the years. Factors responsible for the spread—such as transportation of livestock, stocking density, and bird habitats—have varied greatly from region to region [2]. Hong et al. [3] even argue that the establishment of an effective prevention policy must be broken down by region. Little research examines the role of spatial characteristics of HPAI spread at the county level since its first detection in Korea. The objective of this paper is to determine the effects of various regional characteristics on the spread of HPAI. Specifically, we determine whether there is an inter-county relationship with respect to the occurrence of HPAI during the 2016–2017 outbreak in Korea. In addition, we examine factors that affect the spread of HPAI to provide policymakers with evidence-based policy insights when enacting regional-specific measures that could effectively mitigate HPAI outbreaks in Korea and other countries.

HPAI was first detected in Korea during the 2003–2004 production season, and since then, outbreaks have occurred approximately every other year. HPAI outbreaks grew modestly at first but have increased in magnitude and severity. During its initial outbreak, 19 farms were affected in 10 counties. A few years later, in 2008–2009, the number of reported farms affected by HPAI increased to 33 and had spread across a wider area encompassing 19 counties. During the 2014–2015 production season, HPAI outbreaks were similar in size and extent with 38 reported farms affected in 19 counties. The largest HPAI outbreak occurred most recently in 2016–2017. During that production season, the number of farms affected by HPAI increased dramatically reaching a total of 383. Moreover, the HPAI outbreak spread to a much larger area, affecting 50 counties, corresponding to a fivefold increase in the number of affected counties, compared to the initial 2003–2004 outbreak [1].

In general, managing disease outbreaks remains a critical challenge to maintaining the economic viability of livestock production over the long term. In 2017, Korean livestock production totaled USD 16.8 billion, accounting for 41.9% of agricultural production and 22% of the total farm income from agricultural enterprises [1]. Livestock production, including cows, pigs, milk, eggs, chickens, and ducks, all rank among the top 10 agricultural products in Korea. Livestock production is considered a future growth industry and is projected to create high-value products well into the 21st Century. However, HPAI remains a challenge for the industry to continue with its projected success, and curtailing the spread of HPAI remains an important step toward minimizing national losses in the livestock industry. Therefore, it is crucial to grasp factors that propagate HPAI to maximize the effectiveness of prevention measures and activities.

Cumulatively, HPAI rapidly spreads as livestock farmers, along with their vehicles, visit infected farms where they make direct and indirect contact with host pathogens [3]. Unmonitored visits enable the movement of the pathogen throughout the transportation network that ultimately is transported back to farms. Spatial diffusion of the disease can be explained in terms of a contact network, which models the movement of pathogens along transportation corridors [4]. Specifically, it is necessary to combine individual defense activities with cooperative ones among counties [5]. With this strategy, strengthening local defense measures administered by county municipalities could be effective at preventing the spread of HPAI. Thus, there is a critical need to identify significant factors that affect the spread of HPAI, particularly those factors that fall within the purview of local municipalities.

Moreover, the dissemination of HPAI is closely related to the geographic and environmental factors of a region. In 2017, the Ministry of Environment concluded that HPAI outbreaks in Korea often overlap with migratory patterns of seasonal bird movements [6]. The study by Ito [7] indicates that the propagation of HPAI is affected by wild birds. In addition, the movement of vehicles carrying livestock is considered a method of HPAI propagation [8]. Previous studies on HPAI outbreaks in Korea in 2014 show that livestock vehicle movements constituted 26.9% of the HPAI infection paths [9]. The Korean government implemented a livestock vehicle registration system in August 2012 to preemptively prevent the spread of HPAI outbreaks. People’s movement in certain areas may spread the HPAI virus to nearby areas. Urbanization may further imply population concentration, which indirectly results in reduced farmland in rural areas. This further increases the density of farming in rural areas and increases the spread of disease among farms. The denser the population is, the greater the likelihood of disease exposure and spreading of HPAI. In addition, it is estimated that the impact on the spreading of HPAI is different because there are differences in the poultry breeding environment. Breeding scale effects and preventive response vary. In particular, aging populations in rural areas have been recently emerging [10] and are anticipated in this study to have a significant effect on HPAI.

In addition, livestock epidemics are potentially affected by climate change. The fifth Intergovernmental Panel on Climate Change (IPCC) suggested that mad cow disease, severe acute respiratory syndrome (SARS), brucellosis, and HPAI are the most common infectious diseases worldwide that may be affected by climate change [11]. Host pathogens and mediators of disease differ in survival and reproduction according to climatic factors such as temperature, precipitation, and humidity. Given the sensitivity of those climatic factors to the spread of pathogenic diseases, it warrants the need to analyze how they affect HPAI outbreaks, and this study takes a step in examining these factors.

## 2. Literature Review

Some studies have attempted to measure the impacts of spatial characteristics on the spread of HPAI in different countries. Fang et al. [12] examined the influence of environmental factors and the spatial distribution of HPAI outbreaks in mainland China. They found that HPAI outbreaks occurred in areas with low air temperature, high relative humidity, and high air pressure and that these outbreaks were strongly associated with migratory corridors of seasonal migratory bird movements. Martin et al. [13] employed bootstrapped logistic regression and boosted regression trees to examine the association between HPAI incidences in China. Seven factors were considered in their study (i.e., domestic chicken and domestic waterfowl population density, the proportion of land covered by rice or surface water, cropping intensity, elevation, and human population density) and mapped them to the spatial distribution of HPAI outbreaks. Chicken breeding density, human population density, and elevation were associated with HPAI outbreaks in domestic poultry. Paul et al. [14] and Paul et al. [15] analyzed anthropogenic factors for the risk of HPAI outbreaks in Thailand in 2004–2005. They found that the spread of HPAI was most influenced by population density and the proximity to highway junctions and large cities, highlighting the critical role of transportation in HPAI movements.

Tran et al. [16] stated that it is necessary to examine the spatiotemporal patterns of HPAI outbreaks to establish effective countermeasures in Vietnam. They used a two-stage procedure to identify factors influencing the occurrence of HPAI, predict the probability of HPAI outbreaks, and map the spatiotemporal distribution of HPAI. They found that HPAI outbreaks were more likely to occur in the months of November–January and April–June, and were associated with higher breeding density, higher monthly average temperature, in addition to lower monthly average precipitation and humidity. A global analysis of the spread of HPAI outbreaks by [17] found that wild migratory bird routes, roads and railways, wetlands, land usage, and coverage were significantly associated with HPAI outbreaks.

A meta-analysis of published literature by [18] found that proximity of infected farms, presence of other livestock on a farm, open water sources, and whether a farm was not disinfected were significantly associated with an increased risk of HPAI infection at a farm. Simultaneously, the incidence of HPAI in surrounding areas was also found to affect the occurrence of the disease in a particular area. That is, the occurrence of HPAI is spatially dependent. However, some previous studies (e.g., [18]) have limitations in analyzing the direct and indirect effects of nearby areas on HPAI outbreaks in terms of econometric methodology. It is rather important to incorporate a spatial econometric method for the analysis of factors influencing the propagation of HPAI. Thus, in this study, we apply a spatial econometric model that considers the spatial dependency of HPAI outbreaks and analyzes the spatial interactions of county-level characteristics with regard to the spread of HPAI. Our study contributes to the existing literature by providing useful empirical and data-driven information for establishing and strengthening localized effective defenses against HPAI outbreaks in Korea and other countries where HPAI outbreaks are prevalent. 

## 3. Materials and Methods

### 3.1. Data

Data for the number of farms affected by HPAI in each county from 16 November 2016 to 12 May 2017 (the seventh HPAI outbreak in Korea) were obtained from the Ministry of Agriculture, Food and Rural Affairs ASF∙AI∙FMD∙BSE homepage https://www.mafra.go.kr/FMD-AI2/2179/subview.do and were accessed on 19 September 2020. Data of poultry type—laying hens, broilers, breeders, and ducks, and poultry numbers—were collected at the county level from the Korean National Statistics for 2016 at https://www.weather.go.kr/weather/climate/past_table.jsp and were accessed on 19 September 2020.

Climate data that included monthly average temperature, precipitation, and relative humidity for each county were collected from the Korea Meteorological Administration for yearly records. For each county, the climate data is the average of the monthly totals obtained in January–December 2016. Geographical and environmental factors investigated were the presence of migratory birds and the number of livestock vehicles registered (registration vehicles include various vehicles that carry livestock, crude oil, animal drugs, feed, livestock manure, and compost as of 2016; these vehicles also include vehicles entering animal husbandry facilities for medical treatment, artificial insemination, consulting, sampling, quarantine, and machine repair [19]). A dummy variable was created to indicate the main habitat of migratory wild birds as assigned by the Korean Ministry of Environment and the Korean Ministry of Oceans and Fisheries. The variable was equal to 1 if the county had migratory bird habitat that the Ministry of Environment designated, and zero otherwise. The number of livestock vehicles registered in each province was collected from the Ministry of Agriculture, Food, and Rural Affairs as of 31 December 2016 (https://www.me.go.kr/home/web/public_info/read.do?pagerOffset=0&maxPageItems=10&maxIndexPages=10&searchKey=all&searchValue=&menuId=10357&orgCd=&condition.deleteYn=N&publicInfoId=149&menuId=10357 and was accessed on 19 September 2020).

Demographic and social factors considered were aging level, population growth percentage, migration, population density, and urbanization rate by county (urbanization rate data are from city planning status of Korea Land and Housing Corporation homepage [20]). We considered population growth rate as the percentage of population growth compared to the previous year, whereas migration was defined as the difference between the number of people that had moved into a region and the number of people that had moved out. As for population density, it was defined as the number of residents per km^2^, while the aging level was considered as the ratio of the population aged 65 or older at the county level [21]. Data used in the spatial models are summarized in Table 1. 

**Figure 1:**
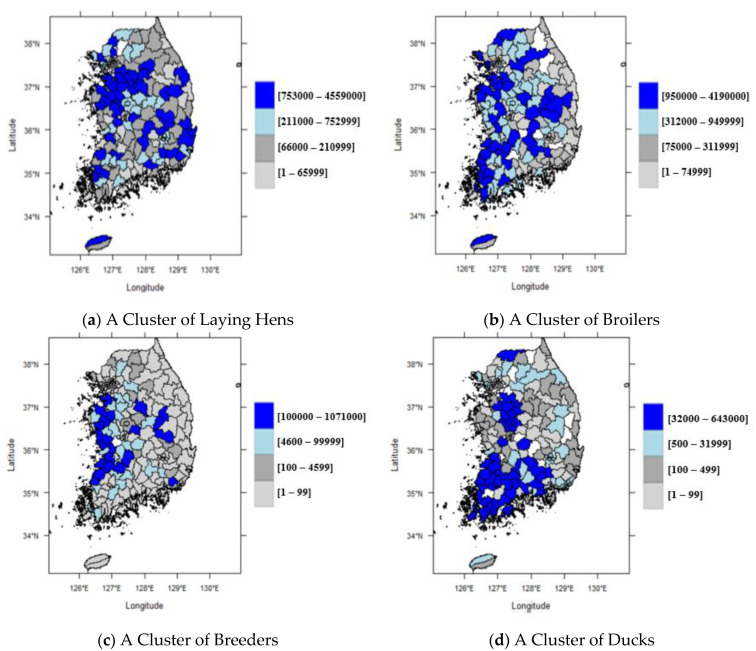
Spatial distribution of poultry numbers in Korea, 2016.

A total of 162 counties were analyzed in this study. Average poultry numbers were 444,260 laying hens, 505,680 broilers, 70,460 breeders, and 36,920 ducks (Table 1). Spatially, poultry production is clustered within certain areas in Korea, as shown in Figure 1.

Climatically, the mean values of temperature, precipitation, and relative humidity for all counties were 13.5 °C, 112.2 mm, and 69.7%, respectively. For the main habitat of migratory birds, the average value across all counties was 0.2. The average number of livestock vehicle registration in all provinces was 301.7. For demographic and social factors, the average population growth rate in 2016 was 0.38%, while the average migration was 0 but ranged from −14,030 to +43,530. The average population density was 1058 people per square kilometer, and the aging index, which represents the proportion of people over 65 years old, averaged 20.6%.

### 3.2. Econometric Procedure

#### 3.2.1. Construction of the Spatial Weight Matrix

One of the most important structural elements of a spatial econometric model is the spatial weight matrix. Being part of the model error structure, a spatial weight matrix is a tool for quantifying and controlling spatial autocorrelation. One of its principal functions is to measure the extent of spatial dependence within the model sphere of influence. Establishing spatial neighbors and measuring the order of their proximity from one another are the two important steps in constructing a spatial weight matrix. A spatial weight matrix can be constructed by a contiguity-based approach, which typically relies on either a versus-distance or N nearest-neighbor approach. Anselin [22] suggests that distance scales are preferable because alternatives, such as the proximity scale, indirectly reflect a spatial arrangement of neighboring elements. Approaches based on distance scale are defined more precisely according to the actual separation in between and spatial arrangement of neighboring elements in the network. Therefore, in this study, a spatial weight matrix by employing the K-nearest-neighbor (KNN) scheme is constructed.

Numerical issues can arise with spatial weight matrices if care is not taken. Specifically, if the spatial weight matrix is populated with the same value throughout the adjacent elements among all of the regions, then there could be significant errors as a result of multicollinearity in the error structure. The spatial weight matrix must, therefore, undergo a row-standardization process, as specified in Equation (1) [23]. Row-standardization quantifies the value of the effects of the surrounding area at one point to give shape to the influence of the surrounding area, as given by Equation (1).

(1) $$W_{ij}^{rs}=\frac{W_{ij}}{\sum W_{ij}},\;\;\sum W_{ij}=k,$$

where *W_ij_* is the spatial weighted value between points *i* and *j* and *k* = 5. The number of nearest neighbors for each county typically ranged from three to five with relatively few counties having >5 neighbors. 

#### 3.2.2. Diagnosis of Spatial Autocorrelation

If spatial autocorrelation is ignored in the analysis, it may bias estimated results [19]. This study tested for spatial autocorrelation using three tests—Moran’s *I* test, the Lagrange Multiplier (LM) test, and Geary’s coefficient. Moran’s *I* index is an indicator of the overall clustering trend in the area under analysis and is estimated by comparing the values of neighboring spatial units. The Moran’s *I* statistic is estimated using the covariance concept shown in Equation (2)

(2) $$\mathrm{Moran}’s\;I=\frac{N}{\sum_{i}\sum_{j}w_{ij}}\frac{\sum_{i}\sum_{j}w_{ij}\left(X_{i}-\bar{X}\right)(X_{j}-\bar{X})}{\sum_{i}{\left(X_{i}-\bar{X}\right)}^{2}},$$

where N is the number of counties, $X_{i}\;\mathrm{and}\;X_{j}$ are the characteristic values of each region, $\bar{X}$ is the average characteristic value of the region in which HPAI occurs, and $w_{ij}$ is the spatial weighted value. Moran’s *I* values are normalized to range between −1 and 1. The closer the Moran’s *I* value is to 1, the more spatially clustered the data are. Alternatively, the closer the value is to −1, the more spatially unclustered the data. A value of zero implies that no correlation or random pattern among neighboring regions exists. The LM test is used to determine a more appropriate spatial regression model and is more specific compared to Moran’s *I* test. Both the LM and Geary’s coefficients were provided by statistical software.

#### 3.2.3. Model Specification

The occurrence and spread of HPAI in a county can be a result of the outbreaks of HPAI in neighboring areas because they share the same spatial characteristics and/or face similar environments. If an ordinary least-squares (OLS) model is estimated to model HPAI occurrence under spatial autocorrelation, the estimated coefficients tend to be inefficient, generating misleading results. To obtain unbiased and consistent estimates for the possible spatial interaction effects of spatial characteristics on the spread of HPAI outbreaks, a spatial econometric model is more appropriate. 

We start with a spatial lag model (SAR) to examine the potential influence of nearby counties on the occurrence of HPAI in a county. In this model specification, we can measure whether the occurrence of HPAI in a specific county depends on the neighboring counties’ HPAI outbreaks. The spatial lag model is expressed as

(3) $$Y=\rho WY+X\beta +\varepsilon ,\;\;\;\varepsilon \sim N\left(0,\sigma^{2}\right),$$

where Y is an N×1 vector of the dependent variable representing the number of occurrences of HPAI in a county, W is an N×N spatial weight matrix describing the spatial proximity between counties, WY denote the endogenous interaction effects among the occurrences of HPAI in nearby counties, and X denotes an N×k matrix of the independent variables that are expected to be associated with the occurrence of HPAI. The parameter ρ represents the spatial autoregressive coefficient that measures the strength of spatial dependence between neighboring counties, β is a vector of parameters to be estimated, and ε is assumed to be an independently and identically distributed error term with zero mean and constant variance $\sigma^{2}$. The reduced form of Equation (3) is

(4) $$Y={\left(I-\rho W\right)}^{-1}X\beta +{\left(I-\rho W\right)}^{-1}\varepsilon ,$$

where each inverse can be expressed as an infinite series, shown in Equation (5).

(5) $${\left(I-\rho W\right)}^{-1}=I+\rho W+\rho^{2}W^{2}+\cdots \approx \frac{1}{1-\rho },\;\;\left(0\leq w_{ij}\leq 1,\;\left|\rho \right|<1\right).$$

The spatial lag model directly reflects spatial autocorrelation among the dependent variables and the regression parameter *β* with a hybrid coefficient given by ${\left(I-\rho W\right)}^{-1}\times \beta$ (Equation (4)). Therefore, the occurrence of HPAI in a specific county implies the change of the characteristics of the county and the influence of the characteristics onto adjacent surrounding counties as governed by the spatial weight matrix. Here, ${\left(I-\rho W\right)}^{-1}$ denotes a spatial multiplier, implying either an indirect or external effect on spatial interactions [23].

If there exists a spatial dependency between the errors in the OLS model, the covariance of these errors increases, and the model becomes inefficient. A spatial error model (SEM) can control the spatial autocorrelation of the errors by assigning a spatial weight matrix in the error terms. The standard form of the spatial error model is presented in Equation (6).

(6) $$Y=X\beta +e,\;\;\;e=\lambda We+\varepsilon ,\;\;\;\varepsilon \;\sim \;N\left(0,\;\sigma^{2}I\right),$$

where We denotes the interaction effects among the error terms of neighboring counties, and λ is the spatial autocorrelation coefficient that measures the strength of spatial dependence between neighboring counties’ unobserved characteristics. As in the spatial error model, Equation (7) can be derived by modifying Equation (6).

(7) $$Y=X\beta +{\left(I-\lambda W\right)}^{-1}e,$$

(8) $${\left(I-\lambda W\right)}^{-1}=I+\lambda W+\lambda^{2}W^{2}+\cdots \approx \frac{1}{1-\lambda },\;\;\;\left(0\leq w_{ij}\leq 1,\;\left|\lambda \right|<1\right),$$

where the matrix ${\left(I-\lambda W\right)}^{-1}$ is the spatial multiplier that can be expressed as an infinite series.

A general spatial model (SARMA) contains spatial dependence in both the dependent variable and the random error terms that account for both endogenous interaction effects and an interaction effect among the error terms. The general spatial model and its reduced form can be expressed as

(9) $$Y=\rho WY+X\beta +\lambda We+\varepsilon ,\;\;\;\varepsilon \;\sim \;N\left(0,\;\sigma^{2}I\right).$$

It is worth mentioning that the general spatial model is appropriate when both spatial interaction effects appear to be statistically significant (ρ≠0 and λ≠0) but reduces to either the SAR model if ρ≠0 and λ=0 or the SEM model if ρ=0 and λ≠0. To estimate these two different types of interaction effects of spatial characteristics on the spread of HPAI occurrences, a maximum likelihood (ML) estimator is used to estimate the models, thereby allowing us to distinguish the explanatory power of the alternative interaction effects. 

### 3.3. Testing the Goodness of Fit of the Model 

To select the most appropriate model among the three competing spatial models (SAR, SEM, and SARMA), we tested the fitness of the models through the bottom-up approach proposed by Anselin and Rey [24]. The bottom-up approach involves firstly estimating the OLS model and then conducting the LM test and the robust LM test to establish the autocorrelation of dependent variables and errors of the OLS model. There are three types of LM test—LM–lag, LM–error, and LM–SARMA. The LM–lag test verifies the spatial autocorrelation between dependent variables, and the LM–error test verifies the spatial correlation between error terms. LM–SARMA is an alternative for a higher-order model of the spatial autoregressive moving average [25]. If the LM statistics are statistically significant in both the spatial lagged and spatial error models, a more statistically significant model is used through the robust LM test. In general, the explanatory power and goodness of fit of the general regression model are often determined by $R^{2}$. However, the spatial regression model yields a pseudo-$R^{2}$ value that is not useful for a spatial model. Accordingly, the values of log likelihood, Akaike information criterion (AIC), and Bayesian information criterion (BIC) are used to determine the goodness of fit of the spatial models. All our analyses were conducted using the R software using the “spdep” package [26,27,28].

## 4. Results and Discussion

Table 2 presents results of the Moran’s *I* test results for spatial autocorrelation. The OLS residuals were used to calculate Moran’s *I* value. As shown in Table 2, Moran’s *I* statistic was 0.2283 and statistically significant at the 1% significance level. This implies that there is strong evidence that HPAI occurrence by county in Korea exhibits spatial autocorrelation. Since spatial autocorrelation of HPAI is confirmed by Moran’s *I* test, it is therefore appropriate to apply the spatial econometric models rather than the OLS model.

To specify the spatial econometric models, the LM and robust LM test statistics were used to determine between the alternative models. The SAR LM–lag and the SEM LM–error values were both statistically significant at the 1% level. The SAR robust LM value was 26.7410 and statistically significant at the 1% significance level. However, the SEM robust LM–error was not statistically different from zero. Based on the LM tests, estimating a spatial econometric model would be more appropriate than a linear model by OLS. For completeness, all three spatial models (SAR, SEM, and SARMA) were estimated.

The AIC and BIC values are used to determine which spatial model best fits the empirical data. The AIC and BIC measures for the SAR, SEM, and SARMA models are 912.371, 930.141, and 911.528, and 915.936, 933.703, and 915.298, respectively. The SAR model and the SARMA model both equally provide the “best fit” based upon both criteria. The estimation results from both SAR and SARMA are comparable since the coefficients have the same direction and magnitude. The SARMA model is more appropriate when both spatial interaction effects are statistically significant (*ρ* ≠ 0 and λ ≠ 0), which is the case, capturing the spatial correlation among the error terms that was negatively associated with HPAI occurrence. 

The estimation results from OLS, SAR, SEM, and SARMA models are presented in Table 3. The regression estimate representing the estimated *ρ* value under the SARMA model is 0.5624. This estimate is statistically significant at the 1% level. This regression coefficient implies that 56.24% of the occurrence of HPAI in a specific area is influenced by infection from neighboring areas. The spatial ripple effect is 2.29 (${\left(I-\rho W\right)}^{-1}=\left(\frac{1}{1-0.5624}\right)=2.2852$). The spatial ripple effect represents the extent to which spatially independent variables affect the spread of HPAI. This value indicates adjacent counties moderately affect the spread of HPAI.

**Figure 2:**
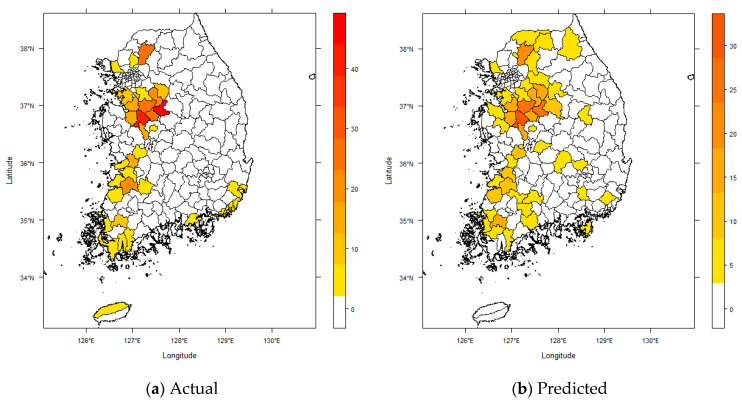
Distribution of the number of farms affected by HPAI (**a**) actual vs. (**b**) predicted using the general spatial model.

The number of farms affected by HPAI averaged 2.59 per county with a range of 0 to 46 HPAI affected farms per county (Figure 2). Figure 2b provides visualizations of the predictions (with uncertainties) made by the SARMA model compared with the actual data in Figure 2a. The results in Figure 2b are comparable with those in Figure 2a, suggesting that the SARMA model’s prediction accuracy was well.

The coefficients for the number of laying hens and ducks are 0.005 and 0.018, respectively, and both are statistically significant at the 1% level. This implies that for larger numbers of laying hens and ducks, the likelihood of the HPAI occurrence increases. The number of ducks has a larger estimated effect on HPAI occurrences. Similar results regarding the significant effect of ducks on the spread of HPAI have been previously reported [29,30,31,32].

Temperature and precipitation were found to be not statistically significant at affecting the spread of HPAI, while relative humidity was significant at the 5% level. The coefficient of relative humidity is −0.109, which indicates that the occurrence of HPAI, on average, increases by about 1 farm/county for every 10% reduction in relative humidity, holding other factors constant. This implies that HPAI is affected by relative humidity and that dry areas are more vulnerable to HPAI. This is consistent with the findings from the analysis of Vietnam’s Red River Delta region where humidity was also found to have a significant and negative effect on the spread of highly pathogenic diseases [16]. 

The number of livestock vehicle registrations is statistically significant at the 1% level. The coefficient on the livestock vehicle registrations is −0.010, which indicates that the HPAI decreases by about 10 farms per county for additional 1000 registered livestock vehicles. This finding suggests that there are concerns regarding the occurrence and spread of disease in provinces where registered livestock vehicles are small.

Population growth rates, population movements, and population densities reflecting regional demographics were not found to be significant. However, in a risk-factor analysis of HPAI in Thailand and China, the increase in population density was found to negatively affect the occurrence of HPAI in each country [13,15]. On the other hand, the coefficient of the aging level is −0.172, which is statistically significant at the 1% level, indicating that the occurrence of HPAI decreases by 0.172 in regions where the aging population is 1% higher. In addition, the level of urbanization that reflects the social characteristics of counties in Korea is also high. Our results indicate that HPAI incidences decrease by 0.043 farms/county in regions where the urbanization level was 1% under the regional average. HPAI affects a greater number of farms in rural areas. This is plausible because livestock are more concentrated in rural areas than in urban cities.

## 5. Conclusions

In this study, the spatial correlation between the occurrence of highly pathogenic avian influenza (HPAI) and characteristics of 162 Korean counties in 2016–2017 was analyzed. This was to understand the spread of HPAI better and improve the efficiency of the defense against HPAI. Our results suggest the existence of spatial dependency among counties with respect to the occurrence of HPAI. A general spatial model (SARMA) that accounts for both the spatial autocorrelation of the spread of HPAI between counties and the correlation among the error terms of neighboring counties was the best model to determine the factors influencing the spread of HPAI. After examining the spatial ripple effect of HPAI, 56.24% of the HPAI occurrence in a particular county was found to be influenced by surrounding counties. Coherent policies implemented in a timely manner to minimize potential sources from spreading HPAI from the surrounding counties are necessary to limit the potential damage.

The effect of regional characteristics of counties on the spread of HPAI was also tested. We found that poultry numbers of laying hens, breeders, and ducks have a statistically significant effect on the spread of HPAI. Therefore, as in [29], increased efforts to improve the raising environment found on those poultry farms are recommended to mitigate and prevent the spread of HPAI. Particularly, we found ducks as the most vulnerable to HPAI. This result suggests that counties intensively breeding ducks should be the focus of surveillance and improved methods of disinfection to mitigate the spread of HPAI during outbreaks.

In terms of the climatic factors, the relative humidity of an area was found to affect the occurrence of HPAI. A current policy is to increase inspections to curb the spread of HPAI when humidity forecasts are favorable for increased HPAI spread. Increasing the number of preliminary inspections based upon humidity forecasts in municipalities located inland, such as Gyeonggi and Chungchung Provinces, may prevent or retard HPAI outbreaks since these areas are more susceptible to the effects of humidity than coastal areas. 

For livestock vehicle registrations, our results indicated that the spread of HPAI is less likely to occur in provinces with a larger number of livestock vehicle registrations. The coefficient of livestock vehicles is expected to be positive because livestock vehicles potentially mediate how HPAI spreads [9]. However, in the present study, livestock vehicle registrations were found to be negatively correlated with HPAI outbreaks. This may be because the data for livestock vehicle registration were not county level. It was at a provincial level and might not have captured the actual movements of the vehicles at the county level and could include vehicles not necessarily involved in the poultry industry. For example, there are no HPAI occurrences in Gyeongsang Province, where the number of registered livestock vehicles is the largest, while there are numerous HPAI occurrences in Chungchung Province where there are fewer registered livestock vehicles.

The influence of aging by region reveals that the higher the aging level, the lower the likelihood of the occurrence of HPAI. Whether or not this is a result of breeding practices of elderly farmers remains to be determined. HPAI is more common in rural areas where urbanization is low. Therefore, to promptly prevent the spread of HPAI, we suggest that there is a need to reinforce prevention-centered activities/measures by focusing on rural areas and counties with lower urbanization levels.

This study provides important insights that may be useful for county-specific preventive measures for HPAI outbreaks by estimating the spatial ripple effect of various characteristics at the county level. This study also estimated the spatial dependence of the spread of HPAI across Korean counties. Since we did not have access to panel data that could account for unobserved spatial heterogeneity, this is an important caveat. In addition, our analysis is restricted to the 2016–2017 outbreak, which was more damaging, and not the previous ones that occurred in Korea. This may limit the generalizability of our findings. To build upon our results, future studies should employ panel datasets and incorporate other outbreaks of the HPAI to identify spatial and temporal effects of HPAI outbreaks in Korea and other countries.

## Figures and Tables

**Table 1:** Descriptive statistics (2016, N = 162).

Variables		Mean	Std. Dev.	Max.	Min.
Dependent variable	Farms affected by HPAI (N/county)	2.59	6.82	46.00	0.00
Poultry number (1000 head)	Laying hens	444.26	743.71	4558.81	0.00
	Broilers	505.68	737.57	4190.00	0.00
	Breeders	70.46	156.12	1070.05	0.00
	Ducks	36.92	94.30	642.59	0.00
Poultry Farms	Farm Number (N)	43.54	32.55	197.00	1.00
Climate	Temperature (°C)	13.52	1.28	17.00	8.10
	Precipitation (mm)	112.16	88.66	1056.00	60.49
	Humidity (%)	69.69	5.98	91.90	56.70
Geographic and environmental	Migratory birds (0 or 1)	0.20	0.40	1.00	0.00
	Livestock vehicles (N)	301.70	212.56	845.94	6.75
Demographic and sociological	Population growth (%)	0.38	2.98	26.40	−0.52
	Migration (1000)	0.00	12.81	43.53	−14.03
	Population density (N/km^2^)	1058.00	2474.00	16,408.00	19.00
	Aging level (%)	20.55	8.24	37.49	7.51
	Urbanization (%)	65.06	25.75	100.00	19.06

**Table 2:** Moran’s *I* statistics.

Moran’s *I*	Z-Score	Expectation	Variance	*p*-Value
0.2283	5.1670	−0.0062	0.0021	0.0001

**Table 3:** Estimation results for highly pathogenic avian influenza (HPAI) outbreaks.

Independent Variable	OLS Model	Spatial Lag Model	Spatial Error Model	General Spatial Model
Constant	15.700 **	12.499 **	14.815 **	11.376 **
	(6.342)	(5.327)	(7.217)	(4.646)
Laying hens	0.006 ***	0.005 ***	0.005 ***	0.005 ***
	(0.001)	(0.001)	(0.001)	(0.001)
Broilers	0.002 ***	0.001	0.001	0.001
	(0.001)	(0.001)	(0.001)	(0.001)
Breeders	0.004	0.004 *	0.005 *	0.004 *
	(0.003)	(0.002)	(0.003)	(0.002)
Ducks	0.023 ***	0.020 ***	0.021 ***	0.018 ***
	(0.004)	(0.004)	(0.004)	(0.003)
Poultry farms	0.052 **	0.040 **	0.042 *	0.033 *
	(0.023)	(0.019)	(0.022)	(0.018)
Temperature	0.239	0.081	−0.028	0.130
	(0.291)	(0.244)	(0.312)	(0.212)
Precipitation	−0.005	−0.004	−0.006 *	−0.002
	(0.004)	(0.004)	(0.004)	(0.003)
Relative humidity	−0.107 *	−0.099 *	−0.082	−0.109 **
	(0.063)	(0.053)	(0.066)	(0.047)
Migratory birds	1.042	1.300 *	1.310	1.221 *
	(0.916)	(0.768)	(0.859)	(0.702)
Livestock vehicles	−0.016 ***	−0.011 ***	−0.011 ***	−0.010 ***
	(0.004)	(0.003)	(0.004)	(0.003)
Population growth	0.078	0.032	0.041	0.004
	(0.142)	(0.120)	(0.129)	(0.111)
Migration	0.000	0.000	0.000	0.000
	(0.000)	(0.000)	(0.000)	(0.000)
Population density	0.000	0.000	0.000	0.000
	(0.000)	(0.000)	(0.000)	(0.000)
Aging level	−0.313 ***	−0.201 ***	−0.229 ***	−0.172 ***
	(0.077)	(0.066)	(0.073)	(0.063)
Urbanization	−0.066 ***	−0.047 **	−0.050 **	−0.043 **
	(0.025)	(0.021)	(0.022)	(0.020)
ρ	—	0.463 ***	—	0.562 ***
		(0.070)		(0.079)
Λ	—	—	0.570 ***	−0.343 *
			(0.084)	(0.183)
Adj. *R*^2^	0.612	—	—	
Log likelihood	−455.910	−439.187	−448.071	−437.764
AIC	943.819	912.371	930.141	911.528
BIC	947.171	915.936	933.703	915.299

Notes: *, **, *** represent statistical significance at the 10%, 5%, and 1% levels. Standard errors are shown in parenthesis.

## Data Availability

Data are available upon reasonable request from the corresponding author.
